# EXPOSING THE REALITIES OF THE WORKFORCE IN NURSING HOMES

**DOI:** 10.1093/geroni/igac059.229

**Published:** 2022-12-20

**Authors:** Christine Mueller

**Affiliations:** University of Minnesota, Roseville, Minnesota, United States

## Abstract

For decades, staffing in nursing homes has been highlighted as being inadequate and directly affecting the quality of care received by residents. The COVID-19 pandemic, occurring during the committee’s deliberations, further highlighted and exacerbated the multiple workforce challenges in nursing homes. The committee’s recommendations related to the nursing home workforce will be presented. Dr. Christine Mueller, a professor at University of Minnesota and with expertise in staffing and quality of care, will discuss strengthening the workforce to meet complex and varied resident needs that align with the committee’s recommendations.

